# Rate-dependent left bundle branch block caused by hyperkalaemia

**DOI:** 10.1007/s12471-015-0795-1

**Published:** 2016-01-07

**Authors:** N. E. Fransen, L. de Vos, H. A. M. van Kesteren

**Affiliations:** 1 Department of Emergency Medicine, St. Elisabeth Hospital and TweeSteden Hospital, Tilburg, The Netherlands; 2 Department of Emergency Medicine, TweeSteden Hospital, Tilburg, The Netherlands; 3 Department of Cardiology, TweeSteden Hospital, Tilburg, The Netherlands

A 73-year-old male presented to the emergency department with vomiting and diarrhoea. No abnormalities were found on physical examination, besides a blood pressure of 80/40 mmHg. The blood tests showed acute renal insufficiency and a potassium of 9.0 mmol/L (3.5–5.0). The electrocardiogram (ECG) showed atrial fibrillation and the occurrence of a left bundle branch block (LBBB) when the heart rate exceeded 76 beats per minute (Fig. 1). When the heart rate decreased the ECG abnormalities disappeared (Fig. 2). Previous ECGs had never shown LBBB.

**Fig. 1:**
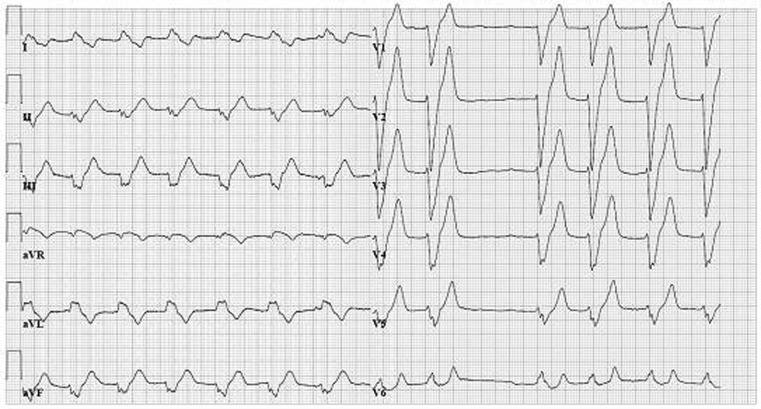
Atrial fibrillation and left bundle branch block

**Fig. 2:**
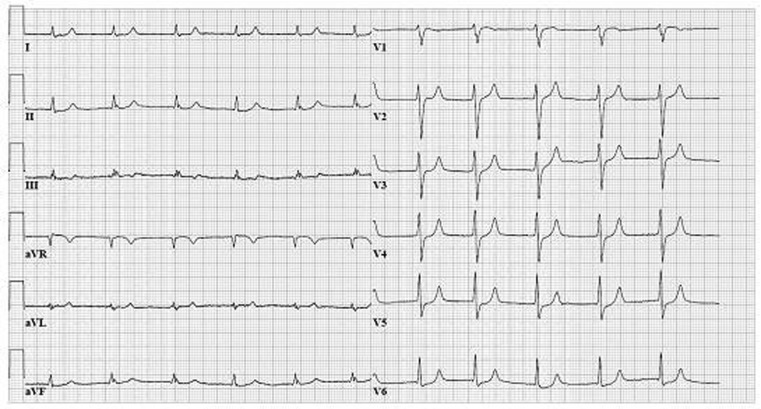
Narrow ECG complexes, the left bundle branch block has disappeared

## Discussion

Although different hyperkalaemia-induced blocks have been described [1], a rate-dependent block has only been mentioned once [2].

Extremely high levels of serum potassium are almost always associated with the classic ECG manifestations; reports of severe hyperkalaemia without these findings are scarce [3]. In our patient, when the heart rate was low, the ECG showed hardly any changes due to the hyperkalaemia. However, when the heart rate increased, clear changes arose, including LBBB.

In the work-up of chest pain in patients with LBBB, all non-invasive techniques fall short in diagnostic accuracy, although prognosis in case of a normal single-photon emission computed tomography (SPECT) does not seem to be altered by LBBB. However, the cardiac event rate of patients with high-risk SPECT is significantly higher than expected from data of patients without LBBB [4, 5].

## Conclusion

The clinician should be aware that the ECG hardly shows any changes due to hyperkalaemia when the heart rate is low, but clear changes can occur with an increasing heart rate.
